# Development of a genetic risk score to predict the risk of hypertension in European adolescents from the HELENA study

**DOI:** 10.3389/fcvm.2023.1118919

**Published:** 2023-06-01

**Authors:** Gloria Pérez-Gimeno, Miguel Seral-Cortes, Sergio Sabroso-Lasa, Luis Mariano Esteban, Empar Lurbe, Laurent Béghin, Frederic Gottrand, Aline Meirhaeghe, Manon Muntaner, Anthony Kafatos, Dénes Molnár, Catherine Leclercq, Kurt Widhalm, Mathilde Kersting, Esther Nova, Diego F. Salazar-Tortosa, Marcela Gonzalez-Gross, Christina Breidenassel, Kathrin Sinningen, Thaïs De Ruyter, Idoia Labayen, Azahara I. Rupérez, Gloria Bueno-Lozano, Luis A. Moreno

**Affiliations:** ^1^ Growth, Exercise, NUtrition and Development (GENUD), Research Group, Instituto Agroalimentario de Aragón (IA2), Instituto de Investigación Sanitaria Aragón (IIS Aragón), Universidad de Zaragoza, Zaragoza, Spain; ^2^ Centro de Investigación Biomédica en Red de Fisiopatología de la Obesidad y la Nutrición (CIBERObn), Instituto de Salud Carlos III, Madrid, Spain; ^3^ Genetic and Molecular Epidemiology Group (GMEG), Spanish National Cancer Research Centre (CNIO), Madrid, Spain; ^4^ Escuela Politécnica de La Almunia, Universidad de Zaragoza, Zaragoza, Spain; ^5^ INCLIVA Biomedical Research Institute, Pediatric Department, Consorcio Hospital General, University of Valencia, Valencia, Spain; ^6^ Université Lille, Inserm, CHU Lille, INFINITE—Institute for Translational Research in Inflammation, Lille, France; ^7^ Risk Factors and Molecular Determinants of Aging-Related Diseases (RID-AGE), Centre Hosp. Univ Lille, Institut Pasteur de Lille, Université de Lille, Lille, France; ^8^ Department of Social Medicine, Preventive Medicine and Nutrition Clinic, University of Crete School of Medicine, Heraklion, Greece; ^9^ Department of Pediatrics, University of Pecs, Pecs, Hungary; ^10^ INRAN, National Research Institute for Food and Nutrition, Food and Nutrition Research Centre-Council for Agricultural Research and Economics, Rome, Italy; ^11^ Division of Clinical Nutrition and Prevention, Department of Pediatrics, Medical University of Vienna, Vienna, Austria; ^12^ Departement of Nutrition—Human Nutrition, Rheinische Friedrich-Wilhelms-Universität Bonn, Bonn, Germany; ^13^ Department of Metabolism and Nutrition, Institute of Food Science and Technology and Nutrition (ICTAN), CSIC, Madrid, Spain; ^14^ Department of Ecology and Evolutionary Biology, University of Arizona, Tucson, AZ, United States; ^15^ PROFITH ‘PROmoting FITness and Health Through Physical Activity’ Research Group, Sport and Health University Research Institute (iMUDS), University of Granada, Granada, Spain; ^16^ ImFine Research Group, Department of Health and Human Performance, Facultad de Ciencias de la Actividad Física y del Deporte-INEF, Universidad Politécnica de Madrid, Madrid, Spain; ^17^ Research Department of Child Nutrition, University Hospital of Pediatrics and Adolescent Medicine, St. Josef-Hospital, Ruhr-University Bochum, Bochum, Germany; ^18^ Department of Public Health and Primary Care, Ghent University, Ghent, Belgium; ^19^ Department of Health Sciences, Institute for Innovation & Sustainable Food Chain Development, Public University of Navarra, Pamplona, Spain

**Keywords:** blood pressure, genetic risk score, adolescents, Europe, prevention, hypertension

## Abstract

**Introduction:**

From genome wide association study (GWAS) a large number of single nucleotide polymorphisms (SNPs) have previously been associated with blood pressure (BP) levels. A combination of SNPs, forming a genetic risk score (GRS) could be considered as a useful genetic tool to identify individuals at risk of developing hypertension from early stages in life. Therefore, the aim of our study was to build a GRS being able to predict the genetic predisposition to hypertension (HTN) in European adolescents.

**Methods:**

Data were extracted from the Healthy Lifestyle in Europe by Nutrition in Adolescence (HELENA) cross-sectional study. A total of 869 adolescents (53% female), aged 12.5–17.5, with complete genetic and BP information were included. The sample was divided into altered (≥130 mmHg for systolic and/or ≥80 mmHg for diastolic) or normal BP. Based on the literature, a total of 1.534 SNPs from 57 candidate genes related with BP were selected from the HELENA GWAS database.

**Results:**

From 1,534 SNPs available, An initial screening of SNPs univariately associated with HTN (*p* < 0.10) was established, to finally obtain a number of 16 SNPs significantly associated with HTN (*p* < 0.05) in the multivariate model. The unweighted GRS (uGRS) and weighted GRS (wGRS) were estimated. To validate the GRSs, the area under the curve (AUC) was explored using ten-fold internal cross-validation for uGRS (0.802) and wGRS (0.777). Further covariates of interest were added to the analyses, obtaining a higher predictive ability (AUC values of uGRS: 0.879; wGRS: 0.881 for BMI *z*-score). Furthermore, the differences between AUCs obtained with and without the addition of covariates were statistically significant (*p *<* *0.05).

**Conclusions:**

Both GRSs, the uGRS and wGRS, could be useful to evaluate the predisposition to hypertension in European adolescents.

## 1. Introduction

During the last decades there has been an increase in hypertension (HTN) prevalence throughout the world, both in children and adolescents (1). A recent meta-analysis found higher HTN prevalence (9.12%) in children older than 10 years, compared with children 6–9 years old (4.06%) (1). The increase in HTN prevalence has been related to other cardiometabolic disturbances such as obesity and dyslipidemia (2, 3), as some physiological mechanisms are shared (4). However, the etiology of essential HTN has not been fully elucidated (5).

The absence of symptoms together with the lack of time during routine outpatient visits have become a challenge for the early HTN detection and diagnosis (6). In addition, adolescents with HTN often remain hypertensive even in adulthood with possible irreversible consequences on their health status (7). In order to prevent future health complications, the American Academy of Pediatrics (AAP) updated in 2017 the Clinical Practice Guideline for Screening and Management related to high BP in children and adolescents. Fixed cut-off points independent of age, sex and height were established for children over 13 years. HTN was defined when BP levels are greater than or equal to 130 mmHg for systolic BP (SBP), or greater than or equal to 80 mmHg for diastolic BP (DBP) (8).

Several risk factors affecting BP levels, such as dietary habits, salt intake, excess adiposity, low levels of physical activity, sleep quality and secondhand smoking, can be modified (9, 10), while others, including genetics, seem to remain unmodified (11). Therefore, early identification of risk factors, not only environmental but also genetics, is essential for the prevention of HTN-related future cardiovascular events (11).

HTN is a highly heritable trait, almost 40%–60% in individual differences in BP have a genetic basis (12). Since the first genome-wide association studies (GWAS) with significant results in 2009, to present a large number of genetic variants associated with BP have been discovered (13). Due to the huge quantity of candidate loci, above 1,000 independent BP genetic signals (14), there is limited potential to carry out the functional investigation of each locus. However, some of them have been fairly well characterized. Some of these plausible candidates are angiotensinogen (*AGT*), Angiotensin-I-Converting Enzyme (*ACE*) and Angiotensin-I-Converting Enzyme 2 (*ACE2*), genes encoding enzymes responsible for the control of the renin-angiotensin system (11). In Chinese populations, most studies have found significant associations between BP and single nucleotide polymorphisms (SNPs) related with these loci (15). Other loci such as ATPase Plasma Membrane Ca^2+^ Transporting 1 (*ATP2B1*) and Calcium Voltage-Gated Channel Auxiliary Subunit Beta 2 (*CACNB2*) genes, that play a critical role in the intracellular calcium homeostasis, have shown several SNPs associated with higher BP (14, 16). Moreover, there are also studies that have found associations between the fat mass and obesity associated gene (*FTO*) and HTN risk, in which BMI plays a moderating role (17).

Although there is a large number of SNPs associated with BP or HTN, each of them independently has a limited predictive ability (18). Thus, the development of a GWAS-based genetic risk score (GRS), to predict the risk of hypertension, could be a useful genetic tool to assess its genetic susceptibility. A GRS is built by combining SNPs, either by summing the number of risk alleles: unweighted GRS (uGRS); or by multiplying the number of risk alleles by each estimated coefficient: weighted GRS (wGRS).

In European adults, several GRS have been associated with BP and HTN (19, 20), also being prognostic markers for future cardiovascular events. For example, a GRS based on 29 genetic variants developed in adults (20) was also analyzed in European children and adolescents (21, 22) Moreover, another study carried out in European adolescents used an adult-based GRS to explain the association with different traits, including BP, in adolescents (23). To date, the most recent GWAS performed in children and adolescents, identifying novel loci associated with BP (24, 25). In addition, to the best of our knowledge, no GRSs have been tested in European adolescents considering GWAS BP-associated loci in both adult population and children and adolescents. Thus, the present study considered loci associated with BP or elevated BP previously found in the literature from adults and adolescents Caucasian populations. Therefore, the aim of the present study was to develop a GRS as a predictive tool for HTN and test it in European adolescents from the “The Healthy Lifestyle in European by Nutrition in Adolescence (HELENA)” cross-sectional study.

## 2. Methods

### 2.1. Study design and population

The HELENA-cross-sectional and multicentric study was conducted in 10 European cities between 2006 and 2007 (Athens, Greece; Dortmund, Germany; Ghent, Belgium; Heraklion, Crete; Lille, France; Pécs, Hungary; Rome, Italy; Stockholm, Sweden; Vienna, Austria; and Zaragoza, Spain) with large enough sample (*N* = 3,528) to represent population diversity (26). The main objective of the HELENA study was to obtain representative data of European adolescents in terms of nutrition, lifestyle and health-related parameters. The HELENA study was implemented following the Declaration of Helsinki 1964 recommendations (revision of 2000) and with the approval of the Ethics Committees of each of the ten participating countries (27). The protocol was approved by the Ethical Committee (Comité de Ética de la Investigación de la Comunidad Autónoma de Aragón: CEICA). Moreover, parents of the participants enrolled in the study read and signed an informed consent. Also, a verbal informed consent was read to the participants whose age was above 16 years old.

A randomly selected sample (Supplementary Figure S1) from the total of participants was assigned for blood sampling (28), selecting a total of 868 adolescents (52.9% females) with complete genetic and BP information, who were not receiving pharmacological treatment for low blood pressure.

### 2.2. Blood pressure assessment

The SBP and DBP were measured twice in sitting position with a 10-minute interval in between, using a previously validated automated digital BP device for clinical use [OMRON M6 (HEM-7001-E)] (29). The blood pressure measure was measured by trained professionals in a quiet room. The cuff-size was adapted to the arm circumference of participant. The complete procedures of BP measurement have been previously described (30). In the present study, the lowest BP measurement, in both SBP and DBP, was selected as the final value. Following the AAP CPG 2017 recommendations, the cut-off points determined in these guidelines were used. The AAP CPG 2017 were created based on children and adolescents with normal weight. Also, unlike ESH, AAP CPG establishes specific cut-off points for adolescents 13 and over. For instance, adolescents aged 13 years or older are classified as having stage 1 HTN when their SBP is at or above 130 mmHg, or their DBP is at or above 80 mmHg (8). Although, three BP reading separated in time are necessary to confirm HTN. In the present study, the term HTN or risk of HTN has been used interchangeably to refer to those participants who had BP levels above or equal the cut-off-points at the time of the BP measurement.

### 2.3. Anthropometric measurements

Weight and height were measured by trained researchers following standardized procedures. BMI was calculated from height and weight (kg/m^2^). Further, the BMI *z*-score was calculated using the recommendations of Cole et al. (31).

### 2.4. Blood collection and genotyping

A standardized methodology for blood collection, transport and analysis was performed by a certified laboratory (32). Blood samples were collected after a 10–12 h overnight fast in EDTA K3 tubes. Then, the DNA was extracted and stored at the Institute of Nutritional and Food Sciences (IEL) of the University of Bonn, and sent to the Laboratoire d'Analyse Genomique Centre de Ressources Biologiques (LAG-CRB) e BB-0033-00071 Institut Pasteur de Lille, F-59000 Lille, France. DNA was extracted from white blood cells with the Puregene kit (QIAGEN, Courtaboeuf, France) and stored at −20°C. Samples were genotyped using the Illumina Global Screening Array chip. After quality control, ∼600,000 genotyped SNPs were available. Additionally, around 7 million SNPs were obtained with imputation using the Haplotype Reference Consortium reference panel. SNPs were excluded if imputation quality was <0.3.

Based on the literature, SNPs related to blood pressure in populations (adults and adolescents) with Caucasian ancestry were selected. The selection criteria for those SNPs found from GWAS was a *p*-value <5 × 10^−8^ (33). All HTN related genes (*n* = 57) encoding these SNPs were acquired from the HELENA genetic dataset and all available SNPs, within these genes, were used to create the GRS.

Among these genes, 13,428 SNPs are available in the HELENA genetic dataset. Unfortunately, the gene *ACE2* was not available due to its location on the X chromosome which was not analyzed in the HELENA study. SNPs quality control Linkage Disequilibrium was calculated via PLINK, in the HELENA genetic dataset, and a *r*^2^ > 0.8 was applied, leading to 4,770 independent SNPs (572 genotyped SNPs and 4,198 imputed) (Supplementary Figure S2).

### 2.5. Development of the genetic risk score

After quality control check, a total of 4,770 SNPs related to BP were obtained from the HELENA GWAS analyses. The SNPAssoc R package was used to establish genetic filters: a Hardy-Weinberg equilibrium (HWE) < 0.05 and minor allele frequency (MAF) > 0.1. A total of 1,534 SNPs were candidates to be included in the GRS. Each SNP was recoded as 0, 1 or 2, depending on the number of risk alleles defined in previous literature, and the additive genetic model was used. Then, using univariate logistic regression analysis with a cut-off *p*-value of 0.1, 166 SNPs were found. Next, a backward/forward stepwise logistic regression algorithm was carried out to select the significant SNPs at *p* < 0.05 (*n* = 16). From the selected SNPs, two methodological approaches were used to create the multivariable GRS, the unweighted method (uGRS) and the weighted method (wGRS). The uGRS was generated by summing the number of risk alleles of each SNP (0, 1 or 2) with a rescaling, for the SNPs that appears as protector factors. The wGRS was the result of multiplying the number of risk alleles at each locus (0, 1, 2) for each estimated coefficient of the multivariate model. Those SNPs that acts as protector factors have a negative coefficient. So when adding them to the score they always subtract in the GRS.

### 2.6. Validation of the genetic risk score

Receiver operating characteristics (ROC) curves analysis was applied to test the diagnostic accuracy of the GRS to classify potential participants with risk of predisposition to HTN or not. For both uGRS and wGRS an area under the curve (AUC) was obtained considering BP as a binary variable. A principal component analysis (PCAs) were calculated on the total sample of SNPs available in order to explain the variability related to ethnicity and center. Additionally, both GRSs were adjusted by PCAs, sex and age, we, from now on we refer to this as GRS baseline.

The models developed were validated internally by 10-fold cross validation. The validation process divided the initial dataset in 10 groups, 9 of them were used to build the predictive model and the last one to validate this model, thus ensuring the validation of models with data not used in their construction.

Moreover, a boxplot was created to graphically show the discrimination ability of the GRS, presenting the distribution of uGRS and wGRS in participants with normal BP and with risk to HTN in a comparison way. Finally, the Youden index was calculated to obtain the best cut-off for the dichotomization of the GRS, and additionally we showed the sensitivity, specificity, negative predictive value, positive predictive value and accuracy for different thresholds for uGRS and wGRS.

### 2.7. Dietary assessment

Dietary intake was determined from two no-consecutive self-administered 24 h recalls at any point in the week. Then, was computerized by a tool previously validated in Flemish adolescents (34): the HELENA Dietary Assessment Tool (HELENA-DIAT). This tool allows participants to select all foods and beverages consumed in six meals (breakfast, morning snacks, lunch, afternoon snacks, dinner and evening snacks). Then, the multiple source method (MSM) was used to calculated individual usual dietary intake. Thus allowing to correct the variability of dietary data between and within participants (35). Additionally, based on the meals salt content reported in the 24 h recalls sodium intake was calculated.

Regarding the assessment of daily fructose intake, the variable created by Béghin et al. (36) in the HELENA study was used. Total fructose exposure was calculated as fructose from monosaccharide form plus fructose from sucrose (0.5 × sucrose per 100 g). The consumption of pure fructose from non-natural foods included food extrinsically fructose rich such as: sugar-sweetened beverages, nonchocolate confectionary, chocolate, cakes/pies/biscuits, desserts and puddings, breakfast cereals and others sources categories (34).

### 2.8. Other covariates

Moderate-to-vigorous intensity physical activity (MVPA) was assessed using an ActiGraph GT1M accelerometer, which has been extensively validated in youth (37). The device methodology was fully described in a previous study (38). Adolescents wore the accelerometer on their lower back for 7 consecutive days, except during water activities. Subsequently, the accelerometry data were downloaded using the Actilife software (Actigraph) with 15 s epochs.

The time spent in moderate and vigorous physical activity (measured in minutes/day) was calculated using on cutoffs of 500 and 1,000 counts per 15 s, respectively. These cut-off point were similar to those used in other study to define activity intensity categories (39).

Birth weight information was obtained through a self-administered questionnaire completed by parents. The questionnaire, along with the information letter and consent forms, was sent to parents and collected at the beginning of the study at school. Parents were specifically asked to recall this information based on their child's health record booklet (30).

### 2.9. Covariate analysis

BMI *z*-score, consumption of pure fructose from non-natural foods, sodium intake, physical activity and birth weight, were added to the two GRSs baseline in order to observe whether the inclusion of other covariates affects GRS predictive ability (uGRS and wGRS). General linear model (GLMs) were performed to evaluate the association between BMI *z*-score, pure fructose, sodium, physical activity and/or birth weight with both uGRS and wGRS. Also, the added predictive ability of covariates was analyzed by significant differences between AUCs using a permutation test, obtained in GRSs with covariates and without them.

### 2.10. Statistical analyses

Anthropometric, BP and pure fructose from non-natural foods consumption variables were descriptively analyzed by sex. Continuous variables (age, height, body weight, BMI, consumption of pure fructose from non-natural foods, SBP and DBP) were reported as median and interquartile range. Differences between groups were analyzed using the Mann–Whitney *U* test.

SBP and DBP were dichotomized. Then, a new HTN variable was created based on whether or not participants had abnormal SBP or DBP values. The newly created BP variable was used as the model outcome. Lastly, the *χ*^2^ test was used to compare BP categories in both sex groups.

All statistical analyses were performed using Rstudio Version 1.2.5019 [*Rstudio Team* (2015)*. RStudio: Integrated Development for R. RStudio, Inc., Boston, MA URL* http://www.rstudio.com/] and the significance level was set at *p* < 0.05.

## 3. Results

### 3.1. Description of the study sample

A total of 869 participants were studied (53% females). Supplementary Table S1 shows the main characteristics of the adolescents participating in the study by sex. All variables showed significant differences except for age, DBP and BMI. A number of 16.7% of the participants showed BP levels above the cut-off points used in our study. Moreover, that percentage was higher in male (11.5%) than in female adolescents (5.2%).

### 3.2. Associations between SNPs and HTN

First, a total of 166 SNPs were associated with HTN in univariate analyses. After the multivariate model developed to build the GRS, the number of SNPs significantly associated with risk to HTN was reduced to 16 (Table 1), correspond to 10 different genes (*STK39*, *ULK4*, *ADRB2*, *SMARCA2*, *PAX5*, *CACNB2*, *PLEKHA7*, *ITGA11*, *FTO* and *UMODL1*). Supplementary Table S1 shows the Linkage Disequilibrium of the 16 SNPs included in the GRS with other SNP located in the same gene. In addition, supplementary Table 2 shows the Linkage Disequilibrium of the 16 SNPs included in the GRS with other SNPs found in the literature. The univariate and multivariate modeĺs odds ratio (OR) were analyzed for each of the SNPs included in the GRS (Table 2). Supplementary Figure S3 shows the direction of each SNP (protective/risk), represented in a forest plot. A number of protector SNPs were obtained: *STK39* rs6433023, *ULK4* rs4580521, *ULK4* rs4973982, *SMARCA2* rs76973157, *CACNB2* rs76466243, *PLEKHA7* rs75351046, *PLEKHA7* rs72865722, *PLEKHA7* rs10832706, *FTO* rs8057044 and *UMODL1* rs113087295. On the other hand, the SNPs that appeared as risk factors of HTN were: *ADRB2* rs17108817, *SMARCA2* rs7048826, *SMARCA2* rs10965093, *PAX5* rs62533676, *ITGA11* rs17320635 and *ITGA11* rs895135.

**Table 1:** Genetic information about the 16 single nucleotide polymorphisms (SNPs) forming the genetic risk score.

rs number	Chromosome loci	Nearest gene	Alleles (major/minor)	MAF	Imputation score	HWE
rs6433023	2:168,856,372	*STK39*	T/C	0.249	0.98477	0.069
rs4580521	3:41,576,183	*ULK4*	A/C	0.346	0.99459	0.1
rs4973982	3:41,710,175	*ULK4*	G/C	0.101	0.99422	0.353
rs17108817	5:148,215,902	*ADRB2*	T/C	0.488	0.94183	0.541
rs76973157	9:2,031,054	*SMARCA2*	A/C	0.117	0.99783	0.870
rs7048826	9:2,143,038	*SMARCA2*	G/C	0.13	0.99179	0.881
rs10965093	9:2,174,253	*SMARCA2*	C/G	0.433	0.9665	0.407
rs62533676	9:36,947,847	*PAX5*	T/G	0.25	0.97257	0.415
rs76466243	10:18,730,310	*CACNB2*	C/G	0.138	0.99581	0.568
rs75351046	11:16,840,511	*PLEKHA7*	C/T	0.244	0.9783	0.927
rs72865722	11:16,947,421	*PLEKHA7*	G/A	0.129	0.9939	0.880
rs10832706	11:16,994,792	*PLEKHA7*	C/T	0.269	0.99972	0.684
rs17320635	15:68,701,651	*ITGA11*	A/G	0.178	0.99943	0.487
rs895135	15:68,723,712	*ITGA11*	G/T	0.361	0.92715	0.339
rs8057044	16:53,812,614	*FTO*	G/A	0.465	0.99549	0.453
rs113087295	21:43,553,681	*UMODL1*	C/T	0.115	0.99787	0.316

*ADRB2*, Adrenergic receptor Beta2; *CACNB2*, Calcium Voltage-Gated Channel Auxiliary Subunit Beta 2; *FTO*, Fat mass and obesity-associated gene; HWE, Hardy-Weinberg Equilibrium; *ITGA11*, Integrin Subunit Alpha 11; MAF, Minor allele frequency*; PAX5*, Paired Box 5; *PLEKHA7*, Pleckstrin Homology Domain Containing A7; *SMARCA2*, SWI/SNF Related, Matrix Associated, Actin Dependent Regulator Of Chromatin, Subfamily A, Member 2; *STK39*, Serine/Threonine Kinase 39; *ULK4*, Unc-51 Like Kinase 4; *UMODL1*, Uromodulin Like 1.

**Table 2:** Association between the risk of hypertension and the 16 single nucleotide polymorphisms included in the genetic risk score, ordered by chromosome number and protector/risk nature.

		Univariate^a^		Multivariate^a^	
rs number	Chromosome	OR (95% CI)	*p*	OR (95% CI)	*p*
rs6433023	2	0.728 (0.557–0.957)	0.021	0.635 (0.470–0.860)	0.003
rs4580521	3	0.689 (0.515–0.914)	0.011	0.593 (0.431–0.807)	0.001
rs4973982	3	0.607 (0.415–0.900)	0.011	0.444 (0.289–0.687)	2.2 × 10^−4^
rs76973157	9	0.669 (0.421–1.022)	0.074	0.534 (0.320–0.855)	0.012
rs76466243	10	0.590 (0.377–0.886)	0.016	0.494 (0.304–0.773)	0.003
rs75351046	11	0.700 (0.507–0.953)	0.027	0.631 (0.447–0.876)	0.007
rs72865722	11	0.722 (0.512–1.032)	0.068	0.484 (0.319–0.735)	6.25 × 10^−4^
rs10832706	11	0.746 (0.554–0.994)	0.050	0.569 (0.401–0.797)	0.001
rs8057044	16	0.688 (0.534–0.884)	0.004	0.624 (0.469–0.826)	0.001
rs113087295	21	0.573 (0.349–0.898)	0.020	0.417 (0.241–0.687)	9.76 × 10^−4^
rs17108817	5	1.310 (1.019–1.690)	0.036	1.491 (1.128–1.982)	0.005
rs7048826	9	1.503 (1.057–2.111)	0.020	1.720 (1.174–2.502)	0.005
rs10965093	9	1.396 (1.087–1.796)	0.009	1.662 (1.256–2.210)	4.17 × 10^−4^
rs62533676	9	1.583 (1.161–2.197)	0.005	1.663 (1.189–2.370)	0.004
rs17320635	15	1.367 (0.991–1.870)	0.053	1.615 (1.136–2.284)	0.007
rs895135	15	1.255 (0.963–1.633)	0.091	1.504 (1.126–2.013)	0.006

^a^ Univariate and Multivariate model of SNP associated with risk of hypertension shown as: Odds Ratio (OR) and 95% confidence interval (CI).

**Figure 1:**
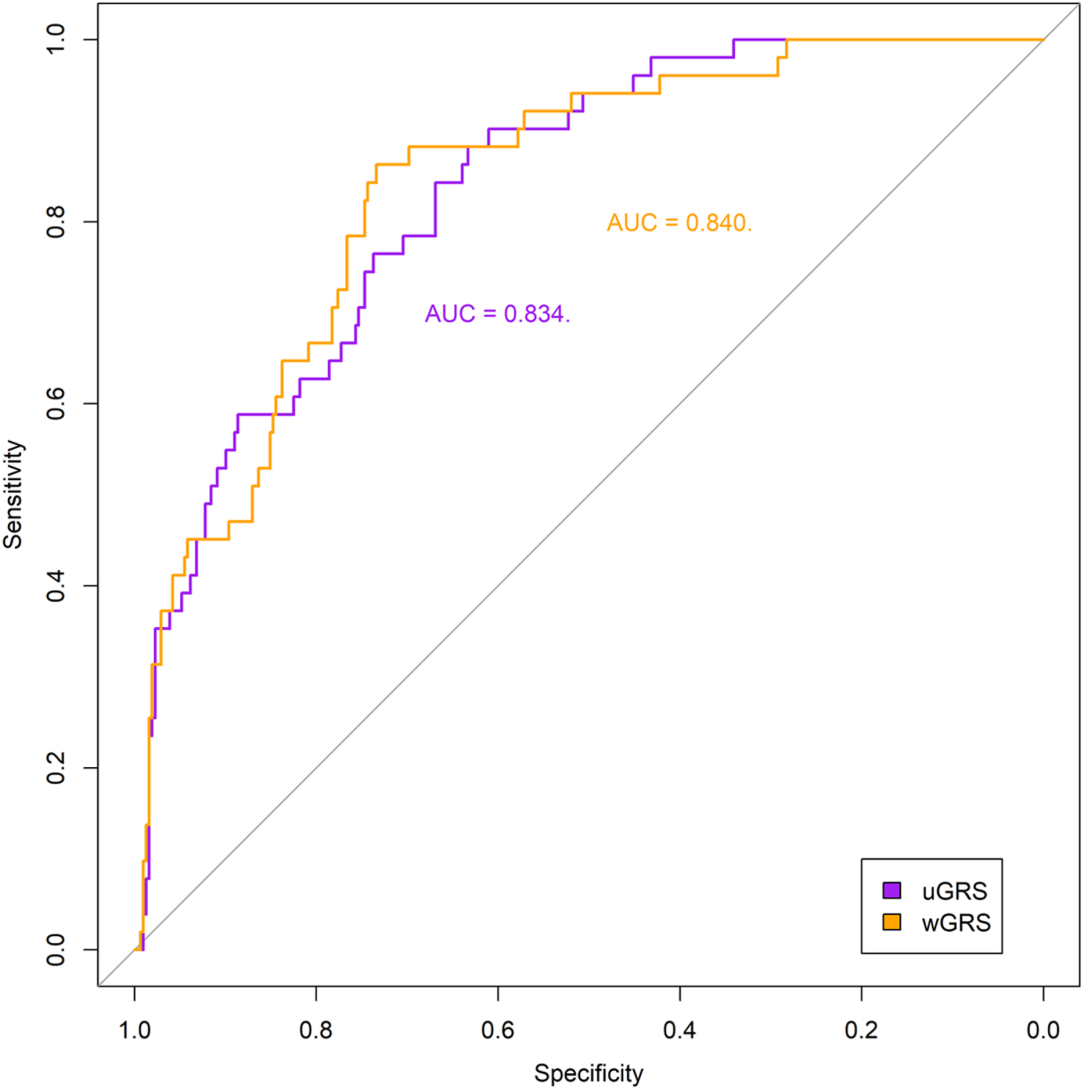
Receiver operating characteristics (ROC) curves of the two genetic risk scores, unweighted (uGRS) and weighted (wGRS), adjusted by principal components analyses, sex and age. Areas under the curves (AUC) are indicated. The straight line represents the ROC expected by chance only.

**Figure 2:**
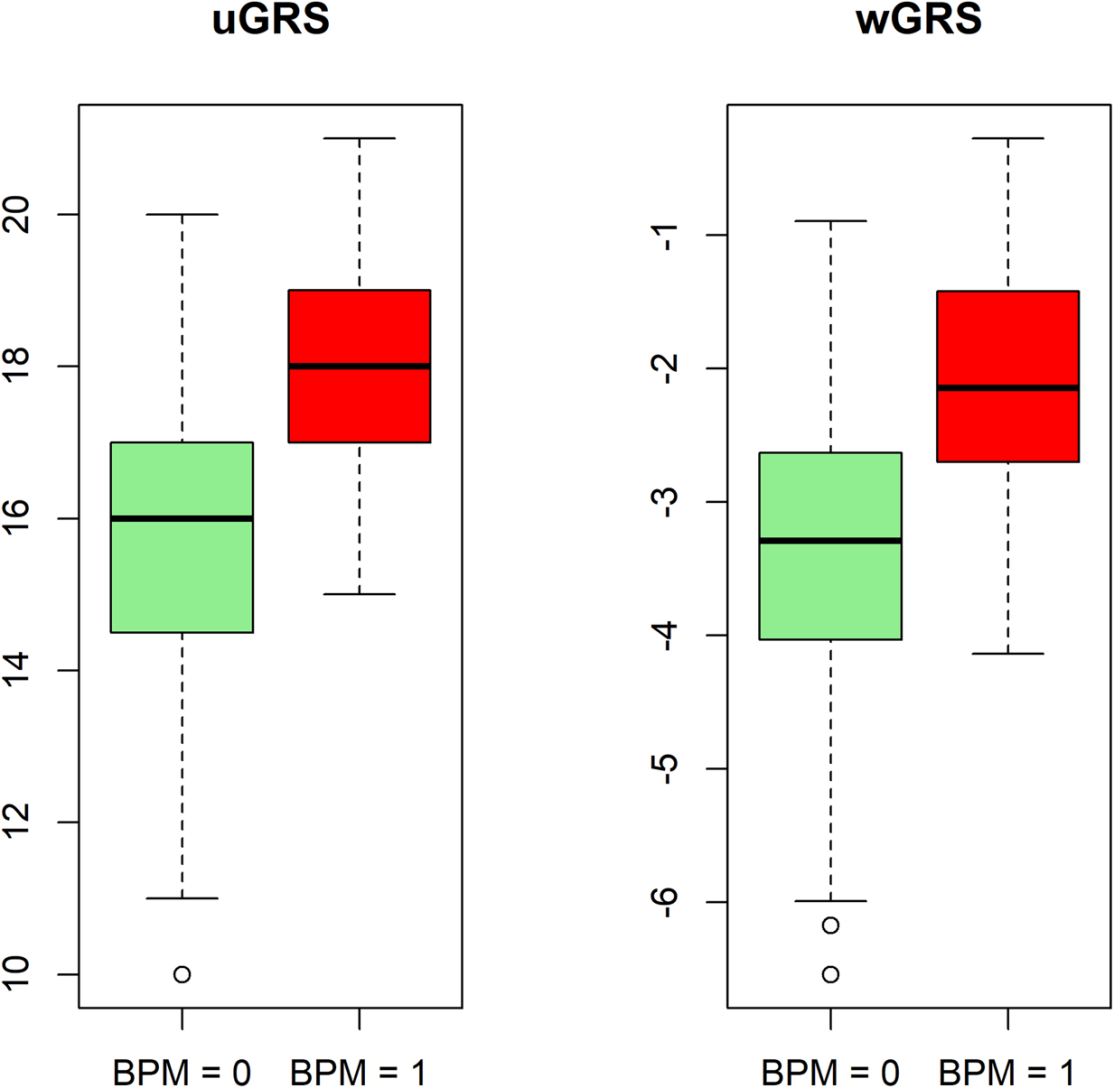
Boxplot of the distribution of unweighted genetic risk score (uGRS) and weighted genetic risk score (wGRS). Values for the groups: normal blood pressure (BPM = 0) and risk of hypertension (BPM = 1).

The predictABEL R package (40) was used to create the uGRS and a wGRS from the multivariate model aiming to predict the risk of HTN. Figure 1 shows the ROC curve and the AUC obtained by comparing the probability of risk of HTN and the real outcome in both GRSs adjusted by PCAs, sex and age (uGRS: 0.834, wGRS: 0.84). The discrimination ability of wGRS and uGRS, adjusted by PCAs, sex and age, was internally validated by 10-fold cross-validation. Both GRSs gives robust predictions (AUC = 0.802 uGRS and AUC = 0.777 wGRS). In addition, a boxplot was created to show the uGRS and wGRS distribution in the different groups, adolescents with normal BP and with risk o HTN (Figure 2). Both GRSs showed differences between the groups. The uGRS showed a possible cut-off point that would differentiate those adolescents with normal BP levels from those with above or equal to the cut-off points established by AAP CPG for HTN. Lastly, the cut-off point with the highest sum of sensitivity and specificity (Youden index) for uGRS was 16.5 and for wGRS was −2.98. Finally, Table 3 shows additional information regarding sensitivity, specificity, positive and negative predictive value and accuracy of the GRS.

**Table 3:** Performance of diagnostic tests in the two GRSs expressed in percentage.

Youden index	Spec	Sens	NPV	PPV	Acc	Youden index	Spec	Sens	NPV	PPV	Acc
uGRS						wGRS					
23	100	0.69	83.41	100	83.43	0.18	100	0.69	83.41	100	83.43
22	99.72	2.07	83.56	100	83.43	0.03	99.86	0.69	83.39	50.00	83.31
21	99.72	3.45	83.76	60.00	83.66	−0.59	99.72	3.45	83.76	71.43	83.66
20	96.69	15.17	85.05	71.43	83.08	−0.90	99.45	5.52	84.01	66.67	83.77
19	90.61	33.10	87.12	47.83	81.01	−1.10	98.76	10.34	84.62	62.50	84.00
18	80.93	55.17	90.02	41.38	76.64	−2.01	88.12	35.86	87.28	37.68	79.40
17	62.30	76.55	92.99	36.70	64.67	−2.68	71.27	64.83	91.01	31.13	70.20
16	44.61	88.28	95.00	28.91	51.90	−2.97	60.91	79.31	93.63	28.89	63.98
15	25.27	96.55	97.34	24.20	37.17	−3.15	55.52	83.45	94.37	27.31	60.18
14	14.09	100	100	20.56	28.42	−3.49	43.23	90.34	95.72	24.17	51.09
13	6.07	100	100	18.90	21.75	−4.02	25.83	97.24	97.91	20.80	37.74
12	1.38	100	100	17.58	17.84	−4.84	10.08	100	100	18.22	25.09
11	0.41	100	100	16.88	17.03	−5.16	5.66	100	100	17.51	21.40
10	0.00	100	100	16.74	16.80	−6.58	0.00	100	100	16.69	16.69

Acc, accuracy; NPV, negative predictive value; PPV, positive predictive value; Sens, sensitivity; Spec, specificity; uGRS, unweighted genetic risk score, wGRS, weighted genetic risk score.

Each row displays the cut-off points of each GRS and the values of each diagnostic test: $\mathrm{Sensitivity}=\frac{\mathrm{True}\;\mathrm{positive}}{\mathrm{True}\;\mathrm{positive}+\mathrm{False}\;\mathrm{negative}}$; $\mathrm{Specificity}=\frac{\mathrm{True}\;\mathrm{negative}}{\mathrm{False}\;\mathrm{positive}}$; $\mathrm{PPV}=\frac{\mathrm{True}\;\mathrm{positive}}{\mathrm{True}\;\mathrm{positive}+\mathrm{False}\;\mathrm{positive}}$; $\mathrm{NPV}=\frac{\mathrm{True}\;\mathrm{negative}}{\mathrm{True}\;\mathrm{negative}+\mathrm{False}\;\mathrm{negative}}$.

### 3.3. Associations between GRS and other covariates

When the BMI *z*-score was added to the GLMs showed a significant association between GRS and BMI *z*-score (*p* < 0.001) both in unweighted and weighted approaches (*β *= 0.834, *β *= 0.818), respectively.

The separate addition of the remaining covariates (pure fructose from non-natural foods, sodium, birth weight and physical activity) to the baseline model did not show significant association with uGRS neither wGRS. Also, the inclusion of pure fructose from non-natural foods to the baseline model plus BMI *z*-score showed significant association for [uGRS (*p* = 0.019) *β *= 0.027 and wGRS (*p* = 0.2) *β *= 0.026 models].

The predictive ability of ROC curves increased when BMI *z*-score was included to the model baseline, obtaining an AUC = 0.879 in the uGRS and AUC = 0.881 in the wGRS (Supplementary Figure S4). Moreover, combining BMI *z*-score and pure fructose in the model baseline, an even greater increase in the predictive ability of the GRSs was observed (uGRS: 0.889, wGRS: 0.891) (Supplementary Figure S5).

In contrast, the addition of sodium, birth weight and physical activity covariates alone to baseline model (uGRS: 0.830, wGRS: 0.838; uGRS: 0.834, wGRS: 0.834; uGRS: 0.834, wGRS: 0.840) (or to the baseline model plus BMI *z*-score (uGRS: 0.880, wGRS: 0.881; uGRS: 0.880, wGRS: 0.882; uGRS: 0.879, wGRS: 0.881) does not affect to predictive capacity of the model (Supplementary Figure S6) and was not significant either (*p* > 0.05).

In addition, significant differences (*p* < 0.05) were observed when comparing the AUC obtained in the GRSs baseline model with that obtained in the model with covariables (baseline + BMI *z*-score + pure fructose from non-natural foods) (*p*(uGRS) = 0.002, *p*(wGRS) = 0.005).

## 4. Discussion

In the present study, an uGRS and a wGRS, considering 16 SNPs associated with BP, were tested in European adolescents. To our knowledge, this is the first GRS performed in European adolescents considering a combination of SNPs significantly associated with BP-related traits both in adults and adolescents Caucasians. Other GRSs have been developed from adults GWAS and then employed in childhood studies (21, 22). In a Finnish follow-up study of children with European ancestry, a weighted GRS was developed, based on 13 SNPs previously associated with BP in adults; the participants with the highest score in the GRS had significant higher diastolic BP at 9 years and the effect was maintained from childhood to adulthood (OR = 1.82) (21). Additionally, a cohort study, evaluated the variance explained by an adult-based GRSs developed for traits related to anthropometry, cardiovascular and renal function, metabolism, and inflammation in Dutch adolescents. For BP levels, the variance explained in adolescents was similar to those observed in adults (23). In contrast, other studies, which analyzed SNPs previously associated with BP in adults, did not find the same associations in adolescents with European ancestry (21, 24) reinforcing the hypothesis that genetic influences on BP change with age (41). The transition from infancy to adulthood comprehends a number of changes in growth and BP. These changes are determined by interactions with age, lifestyle and behavioral risk factors (42). Adolescence is a critical period when individuals with elevated BP levels are more likely to maintain higher BP levels when entering into the adulthood period (43). It is also the stage in which most divergences in BP trajectories are observed (42). For this reason, it is important to know whether there is a difference between the genes that modulate BP in adults and in children. Therefore, for the development of GRSs in adolescents, the SNPs significantly associated with BP found in BP-related GWAS performed in children and adolescents should be considered (24, 25).

There are several genetic polymorphisms involved in metabolic pathways related to BP. In the present study 16 SNPs were included in the GRS. Except for rs8057044, the rest of the SNPs included in the GRS had not been associated with BP in previous studies, despite the previous association between their gene and BP (21, 22, 24, 25).

One SNP was previously associated with obesity, the rs8057044 in the *FTO* gene, that has also been associated with high BP in a study performed in Tunisian adults. Specifically, G carriers of rs8057044 showed an association with high DBP (41). In the present GRSs, the rs8057044 appeared as protective SNP for the development of HTN. This may be explained by the differences in both ethnicity and age between the two studies. Paired box 5 (*PAX5)* genes were also associated with elevated BP in French Canadian adolescents with obesity (12–18 years old) (44). In the mentioned study, those carriers of the rs16933812 showed higher SBP levels. Similarly, in the present study the *PAX5* rs62533676 had a risk role for HTN.

Other SNPs were observed to be associated with BP. For example, the *CACNB2* rs12258967 was analyzed in a study conducted in Lithuanian children and adolescents, showing high odds ratio of high BP in those children with this variant (16). In contrast, in our study, the rs12258967 was not associated with BP in European adolescents. However, another SNP, related to the *CACNB2* rs76466243, was significantly associated with BP and was included in the GRS. There was only one article showing a relationship of the *CACNB2* gene (OR < 1) with diabetic retinopathy (45). Similarly, we have observed an association between this SNP and a protective effect on the development of HTN, (*p* = 0.016).

Another gene related to BP and HTN is the Serine/Threonine Kinase 39 (*STK39*) gene. This gene regulates the Na/Cl cotransporter. Several studies conducted in adult populations have found associations between different *STK39* SNPs and BP or HTN (46). However, a meta-analysis performed in adults from Asian and European populations, which analyzed the most frequent SNPs of the *STK39* gene and their association with HTN did not find conclusive results (47). In children and adolescents the only study found, performed in Chinese children, showed no associations between the most frequent SNP of *STK39* rs3754777 and the risk of HTN (48). Nevertheless, in the GRS developed in this study, a SNP related with the *STRK39* rs6433023 gene has been included. This SNP is a possible protective factor for the development of HTN, hypothesizing that maybe the presence of this SNP could increase the sodium excretion in the renal tubule, reducing the BP levels.

Another widely studied gene has been the Beta 2 adrenergic receptor *(ADRβ2)*; it appears to play an important role in salt-sensitive hypertensive patients (49). In the developed GRS, *ADRβ2* rs17108817 was also a risk factor for the development of HTN.

In addition, genes such as Pleckstrin Homology Domain Containing Family A Member 7 (*PLEKHA7)* and Serine/Threonine-protein kinase (*ULK4)* were found associated with BP (5, 50).However, the SNPs included in that GWAS differ from those obtained in our GRS. In our GRS, all the SNPs associated with these two genes act as a protector factors: rs75351046, rs72865722, rs10832706 for *PLEKHA7* and rs4580521, rs4973982 for *ULK4*.

Interestingly, from GWAS developed in children and adolescents, new loci for BP were identified, such as Integrin Subunit Alpha 11 *ITGA11* rs1563894 associated with SBP assessed in prepuberty, and rs872256 associated with BP during puberty. These loci were not found to be associated with BP in adults. In the GRS developed, two risk *ITGA11* SNPs, rs17320635 and rs895135, were also associated with risk of HTN. Furthermore, in the same GWAS, a SNP (rs872256) near to the **S**WI/SNF-related Matrix-associated, Actin-dependent Regulator Chromatin group A 2 (*SMARCA2*) gene was significantly associated with SBP in puberty children (24). We also found an association between 3 SNPs of the *SMARCA2* gene and BP. Two of them, rs7048826 and rs10965093, are risk SNPs for the development of HTN, whereas rs76973157 had a protective effect.

The GRS developed in the present study showed a good ability to predict the risk of HTN in adolescents. The use of external weights is a practice commonly used in the GRSs development (51). However, in this study, internal weights were used instead. In addition, an uGRS was also built because it is more intuitive to understand than a weighted one. The wGRS and the uGRS, adjusted by principal components analyses, sex and age (baseline model), showed an AUC above 0.8 (uGRS: 0.834, wGRS: 0.84). In the literature, an AUC around or above 0.8 is an accepted value to use for clinical diagnosis (52). In addition, when BMI *z*-score and pure fructose from non-natural foods were added to the GRS, the AUC increased, reaching levels close to 0.9. Both BMI *z*-score and pure fructose from non-natural foods showed a significant association with risk of HTN (*p* < 0.001). BMI and BP are widely related factors and it is expected that they are significantly related. However, the relationship between fructose and BP is controversial (35). A study carried out in European adolescents observed an association between diastolic BP and a high consumption of pure fructose from non-natural foods (36). The relationship between fructose and BP could be explained through the metabolism of this monosaccharide. Fructose is the only carbohydrate that can increases the uric acid levels. This increase causes hemodynamic effects on the organism (increased oxidative stress, endothelial dysfunction and activation of the renin–angiotensin–aldosterone system) and contributes to the BP increase (53).

The present study has some limitations. First, the results should be validated in other populations with larger sample size, similar age and with different ethnic origins. This would allow to test the validity of this BP-specific GRS. Instead, the GRS was internally validated, using cross-validation. Ideally, we should have performed an external validation in an independent cohort. Unfortunately, we did not have the genetic information necessary to carry it out. Second, the HELENA is a cross-sectional study and the long-term effects related with possible cardiovascular complications are not available. Moreover, repeated BP measurements over time were not performed, as suggested by the AAP guideline; therefore, it is not possible to confirm a diagnosis for HTN in this study. In addition, lack of repeated BP measurements over time is one of the reasons why we obtain a high percentage of adolescents with HTN compared with the prevalence observed in other studies. Third, we did not have genetic information of the X chromosome where the *ACE2* gene is located. Perhaps the inclusion of the polymorphisms related to this gene may vary the predictive capacity of the GRS. Fourth, the 24 h recalls assess the sodium content in foods. However, this method does not considered the source of sodium from added salt. According to a recent systematic review a meta-analysis, the monitoring of sodium intake with a 24 h urine is the most accurate method of sodium assessment. Nevertheless, studies developed with high-quality 24 h recalls and in high-income countries showed smaller differences in sodium intake comparing the two methods (54).

In contrast, our study showed some strengths; one was the inclusion of 10 cities from different European countries in the HELENA study. In addition, the GRS developed is formed by several SNPs of genes that have been related to BP in a GWAS performed in children and adolescents, reinforcing the importance of using specific GWAS and GRS for each stage of development. In this line, all SNPs related to all genes found in the scientific literature that are related to BP were included in the analyses, with special interest in those associated with BP in children and adolescents and those previously identified in the adult population. For future research we encourage to validate the present GRS developed in other cohorts with the same genetic ancestries and characteristics in youth.

In conclusion, the GRSs developed in this study could be used as genetic tools to detect adolescents with an increased risk of HTN. Therefore, the BP- specific GRSs may have the potential to guide preventative measures for HTN in youth. Personalized lifestyle interventions, such as diet and physical activity, could target individuals at high genetic risk already from early stages in life.

## Data Availability

The datasets presented in this article are not readily available because of ethics restrictions. Requests to access the datasets should be directed to the corresponding author.
