# Supplementary material for: Development of a genetic risk score to predict the risk of hypertension in European adolescents from the HELENA study
Source: Front Cardiovasc Med. 2023 Jun 1;10:1118919. doi: 10.3389/fcvm.2023.1118919 (PMC10267871; doi:10.3389/fcvm.2023.1118919)
Supplement: Supplementary file 1 [file Datasheet1.docx]

Supplementary Material

# Supplemental Tables and Figures

## Supplementary Table 1. Characteristics of study participants meeting inclusion criteria in the HELENA study.

|  | **Total** | **Males** | **Females** | *p* |
| --- | --- | --- | --- | --- |
|  | n = 868 | n = 409 | n = 459 |  |
| Age (yrs.) | 14.8 (13.8-15.8) | 14.8 (13.8-15.8) | 14.8 (13.8-15.8) | 0.746 |
| Height (cm) | 166.0 (159.2-172.1) | 170.0 (163.0-177.1) | 162.5 (157.4-167.2) | < 0.001 |
| Body Weight (kg) | 59.2 (50.9-65.2) | 62.4 (52.3-70) | 56.3 (49.9-61) | < 0.001 |
| BMI (kg/m^2^) | 21.4 (18.9-23.1) | 21.4 (18.8-23.2) | 21.3 (19.0-23.0) | 0.845 |
| BMI z-score | 0.49 ± 1.1 | 0.62 ± 1.1 | 0.37 ± 1.1 | <0.001 |
| Pure fructose from non-natural foods (g/day) | 20.3 (8.4-27.2) | 24.8 (10.9-32.0) | 16.2 (7.2-22.0) | < 0.001 |
| SBP (mmHg) | 116 (108-125) | 120.0 (112-129) | 112.8 (105-120) | < 0.001 |
| DBP (mmHg) | 65 (59-70) | 64.5 (59-70) | 65.2 (60-70) | 0.260 |
| Blood pressure categories (N,%) |  |  |  | < 0.001 |
| Normal Blood pressure | 723 (83.3) | 309 (42.7) | 414 (57.3) |  |
| Hypertension* | 145 (16.7) | 100 (11.5) | 45 (5.2) |  |

Mann-Whitney-Wilcoxon test was performed to observe differences between sex in non-normal variables. For these variables, the values are presented as median (p25 – p75). For BMI z-score a t-test was implemented and results were expressed such mean ± standard deviation. In addition, chi-square test was performed to observe sex differences between normal blood pressure and hypertension.

*Participants with a BP levels ≥130 mmHg for SBP or/and ≥ 80 mmHg for DBP.

**Supplementary Table 2.** SNPs and Linkage Disequilibrium for the 16 SNPs included in the genetic risk score.

| rs ID | Chromosome | Chromosome position | SNP tested For LD | Linkage Disequilibrium r2 |
| --- | --- | --- | --- | --- |
| rs6433023 | 2 | 2:168856372:T:C |  |  |
| rs4580521 | 3 | 3:41576183:A:C | 3:41710175:G:C | 0.00124266 |
| rs4973982 | 3 | 3:41710175:G:C | X | X |
| rs17108817 | 5 | 5:148215902:T:C |  |  |
| rs7048826 | 9 | 9:2143038:G:C | 9:2174253:C:G | 0.00220405 |
|  |  |  | 9:36947847:T:G | 0.000458139 |
|  |  |  | 9:2031054:A:C | 1.02765e-06 |
| rs10965093 | 9 | 9:2174253:C:G | 9:36947847:T:G | 0.000257395 |
|  |  |  | 9:2031054:A:C | 0.000308038 |
| rs62533676 | 9 | 9:36947847:T:G | 9:2031054:A:C | 4.71506e-05 |
| rs76973157 | 9 | 9:2031054:A:C | X | X |
| rs76466243 | 10 | 10:18730310:C:G |  |  |
|  |  |  | 11:16947421:G:A | 0.00343445 |
|  |  |  | 11:16994792:C:T | 0.0206258 |
| rs72865722 | 11 | 11:16947421:G:A | 11:16994792:C:T | 0.109522 |
| rs10832706 | 11 | 11:16994792:C:T | X | X |
| rs17320635 | 15 | 15:68701651:A:G | 15:68723712:G:T | 0.00047998 |
| rs895135 | 15 | 15:68723712:G:T | X | X |
| rs8057044 | 16 | 16:53812614:G:A |  |  |
| rs113087295 | 21 | 21:43553681:C:T | rs113087295 |  |

LD (r²) is calculated only for SNPs located in the same chromosome.

**Supplementary table 3.** Linkage disequilibrium between 16 SNPs included in the HELENA GRS and SNPs found in the literature.

| Rs ID in the HELENA GRS | Rs ID in the literature | Gene | Chromosome position SNP HELENA | Chromosome position SNP  literature | Linkage Disequilibrium r2 |
| --- | --- | --- | --- | --- | --- |
| rs6433023 | rs3754777 | *STK39* | 2:168856372:T:C | 2:169015914:C:T | 0.0807489 |
| rs4580521 | rs9815354 | *ULK4* | 3:41576183:A:C | 3:41912651:G:A | 0.022229 |
| rs4580521 | rs2272007 | *ULK4* | 3:41576183:A:C | 3:41996136:T:C | 0.0221429 |
| rs4580521 | rs1052501 | *ULK4* | 3:41576183:A:C | 3:41925398:C:T | 0.0229611 |
| rs4580521 | rs3774372 | *ULK4* | 3:41576183:A:C | 3:41877414:T:C | 0.0224663 |
| rs4580521 | rs1716975 | *ULK4* | 3:41576183:A:C | 3:41960006:T:C | 0.023108 |
| rs4580521 | rs9852991 | *ULK4* | 3:41576183:A:C | 3:41875455:C:A | 0.0224663 |
| rs4580521 | rs6768438 | *ULK4* | 3:41576183:A:C | 3:41865355:G:A | 0.0231995 |
| rs4973982 | rs9815354 | *ULK4* | 3:41710175:G:C | 3:41912651:G:A | 0.0136826 |
| rs4973982 | rs2272007 | *ULK4* | 3:41710175:G:C | 3:41996136:T:C | 0.0149245 |
| rs4973982 | rs1052501 | *ULK4* | 3:41710175:G:C | 3:41925398:C:T | 0.0149245 |
| rs4973982 | rs3774372 | *ULK4* | 3:41710175:G:C | 3:41877414:T:C | 0.0137548 |
| rs4973982 | rs1716975 | *ULK4* | 3:41710175:G:C | 3:41960006:T:C | 0.0157458 |
| rs4973982 | rs9852991 | *ULK4* | 3:41710175:G:C | 3:41875455:C:A | 0.0137548 |
| rs4973982 | rs6768438 | *ULK4* | 3:41710175:G:C | 3:41865355:G:A | 0.0149985 |
| rs17108817 | rs1042713 | *ADRB2* | 5:148215902:T:C | 5:148206440:G:A | 0.220417 |
| rs17108817 | rs1042714 | *ADRB2* | 5:148215902:T:C | 5:148206473:G:C | 0.244799 |
| rs76973157 | rs872256 | *SMARCA2* | 9:2031054:A:C | 9:2496480:T:A | 8.93184e-05 |
| rs7048826 | rs872256 | *SMARCA2* | 9:2143038:G:C | 9:2496480:T:A | 0.0026407 |
| rs10965093 | rs872256 | *SMARCA2* | 9:2174253:C:G | 9:2496480:T:A | 1.72956e-06 |
| rs62533676 | rs16933812 | *PAX5* | 9:36947847:T:G | 9:36969205:G:T | 0.0622423 |
| rs76466243 | rs12258967 | *CACNB2* | 10:18730310:C:G | 10:18727959:C:G | 0.0895747 |
| rs75351046 | rs381815 | *PLEKHA7* | 11:16840511:C:T | 11:16902268:C:T | 0.000387122 |
| rs75351046 | rs7926335 | *PLEKHA7* | 11:16840511:C:T | 11:16917869:C:T | 0.000924519 |
| rs75351046 | rs11024074 | *PLEKHA7* | 11:16840511:C:T | 11:16917219:T:C | 0.000492447 |
| rs72865722 | rs381815 | *PLEKHA7* | 11:16947421:G:A | 11:16902268:C:T | 0.384548 |
| rs72865722 | rs7926335 | *PLEKHA7* | 11:16947421:G:A | 11:16917869:C:T | 0.355013 |
| rs72865722 | rs11024074 | *PLEKHA7* | 11:16947421:G:A | 11:16917219:T:C | 0.351055 |
| rs10832706 | rs381815 | *PLEKHA7* | 11:16994792:C:T | 11:16902268:C:T | 0.00678786 |
| rs10832706 | rs7926335 | *PLEKHA7* | 11:16994792:C:T | 11:16917869:C:T | 0.00797906 |
| rs10832706 | rs11024074 | *PLEKHA7* | 11:16994792:C:T | 11:16917219:T:C | 0.006321 |
| rs17320635 | rs1563894 | *ITGA11* | 15:68701651:A:G | 15:68635775:A:G | 0.0283052 |
| rs895135 | rs1563894 | *ITGA11* | 15:68723712:G:T | 15:68635775:A:G | 0.00177236 |
| rs113087295 | rs220299 | *UMODL1* | 21:43553681:C:T | 21:43502762:C:T | 0.00454606 |

LD (r²) is calculated between the 16 SNPs and the ones associated with the same gene in the literature.

Supplementary figure 1. Sampling and recruitment process of the HELENA study.


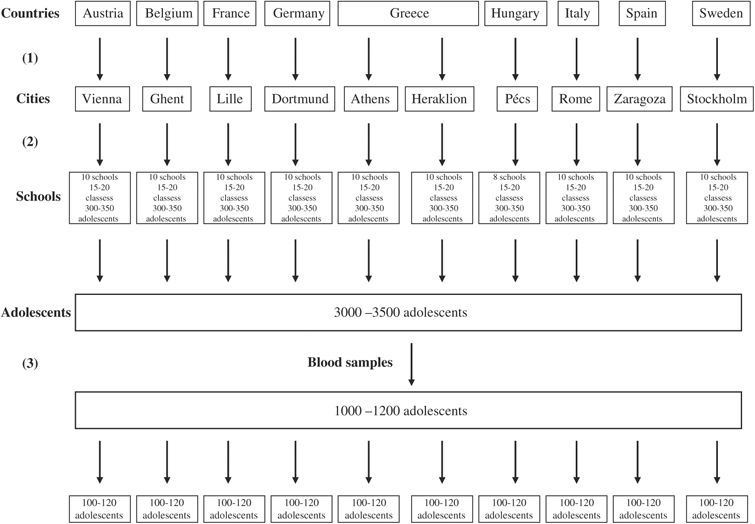


(3) Randomly selection for blood sampling.

Supplementary figure 2. Flow-chart of those single nucleotide polymorphism that are part of the genetic risk score.

**Supplementary figure 3.** Forest plot of single nucleotide polymorphisms (SNPs) negatively (OR<1) and positively (OR>0.1) associated with risk of hypertension. Legends: Protective SNPs against risk of hypertension are shown in the upper part of the forest plot, and SNPs with risk predisposition to hypertension are shown in the bottom part. Multivariate model Odds Ratio (O.R) and 95% confidence intervals (C.I.) displayed.

**
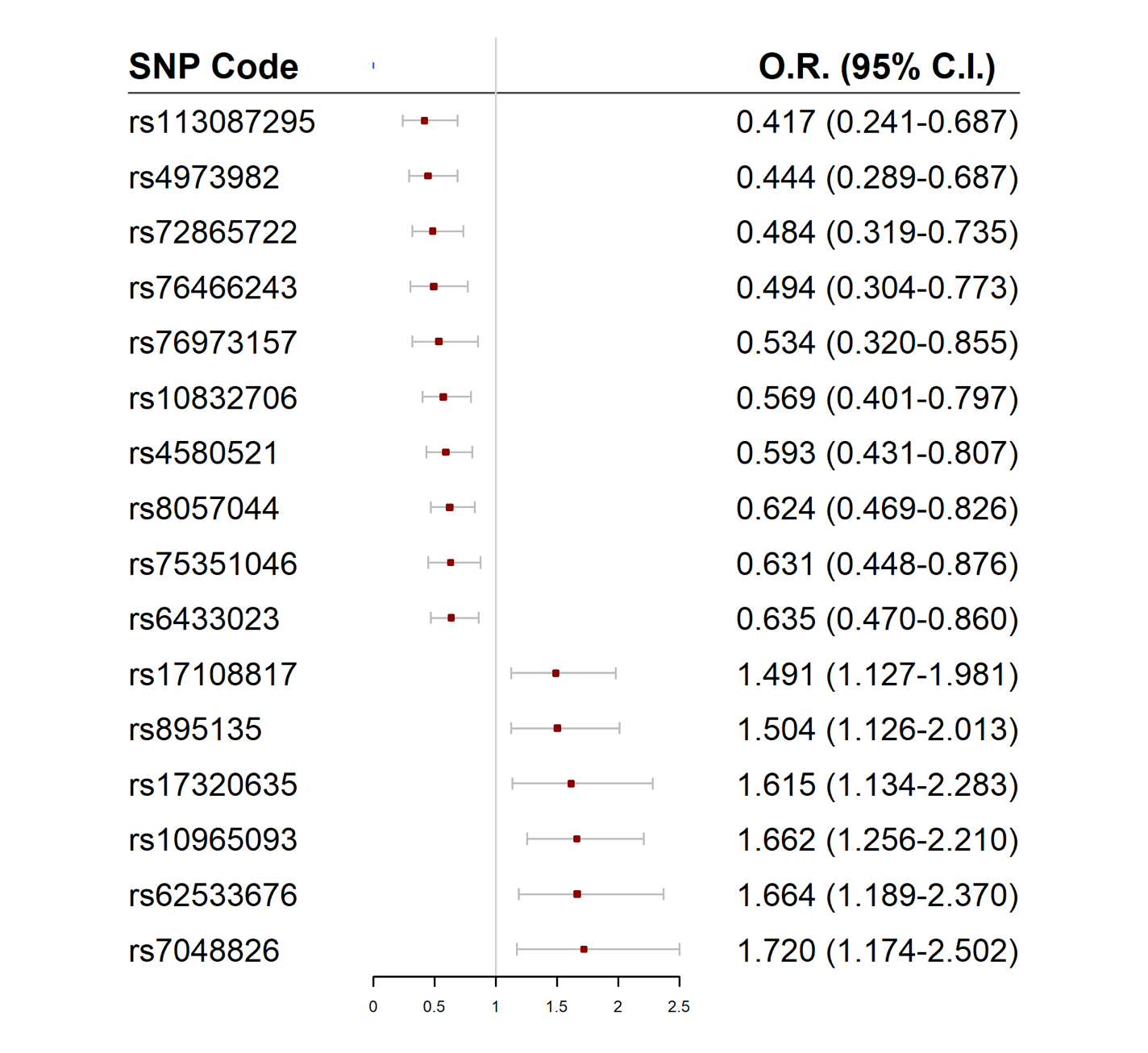
**

**Supplementary figure 4**. Receiver operating characteristics (ROC) curves of the unweighted (uGRS) and weighted (wGRS) genetic risk scores adjusted by principal components analyses, sex and age with BMI-z score added to the model. Areas under curves (AUC) are indicated. The straight line represents the ROC expected by chance only


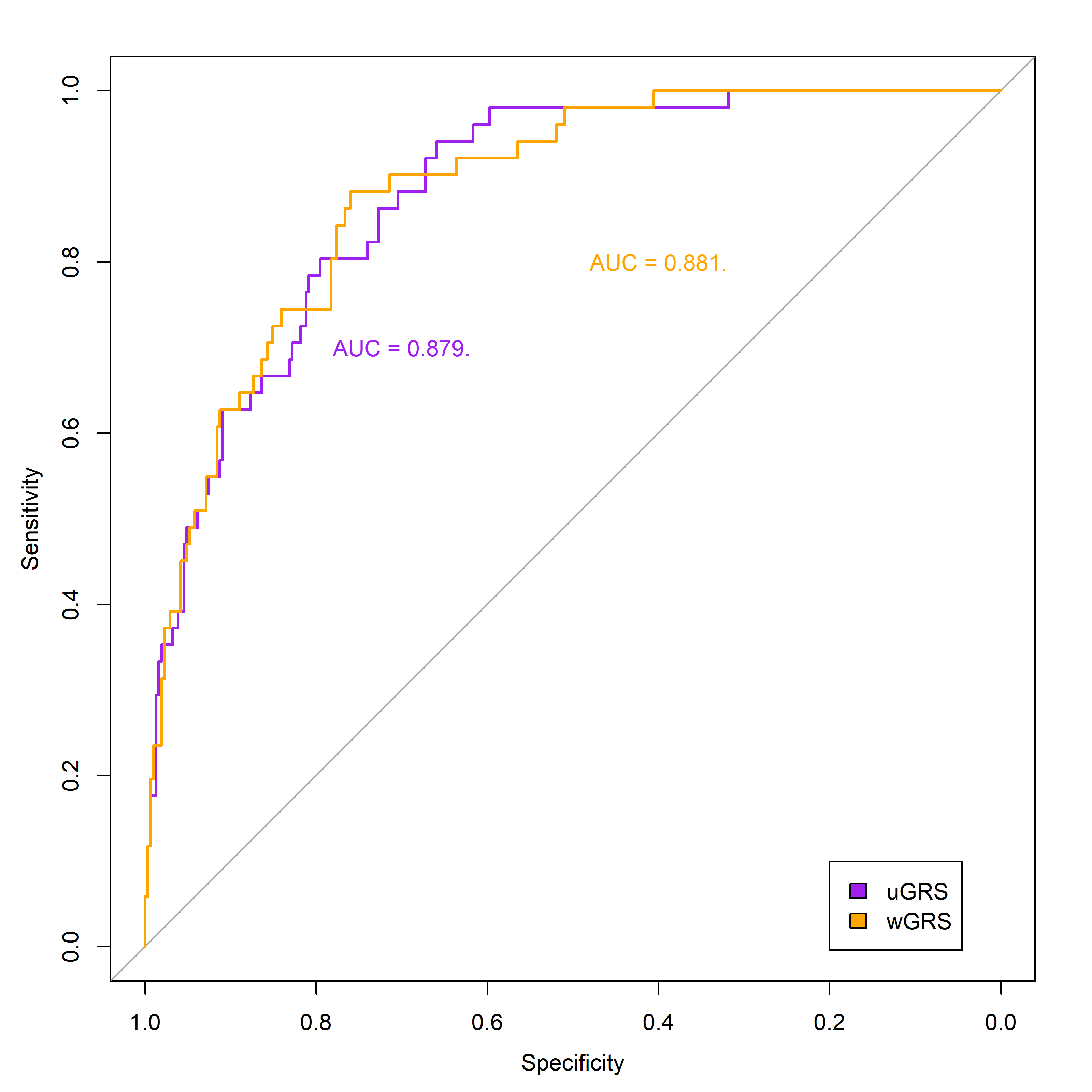


**Supplementary figure 5**. Receiver operating characteristics (ROC) curves of the unweighted (uGRS) and weighted (wGRS) genetic risk scores adjusted by principal components analyses, sex and age with BMI-z score and pure fructose from non-natural foods added to the model. Areas under curves (AUC) are indicated. The straight line represents the ROC expected by chance only.


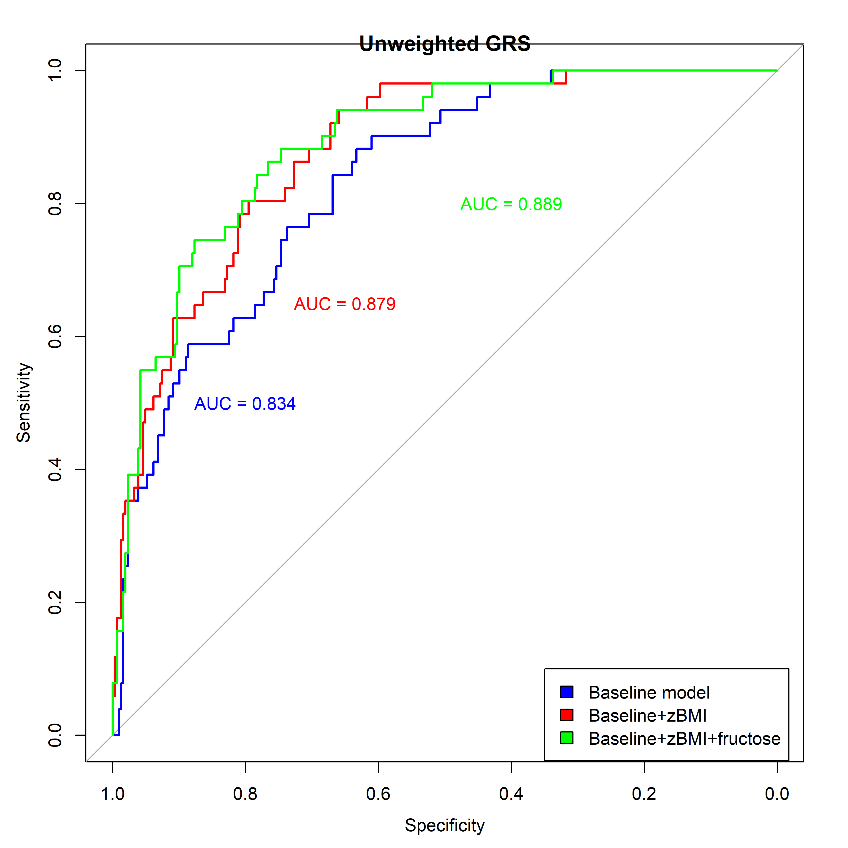

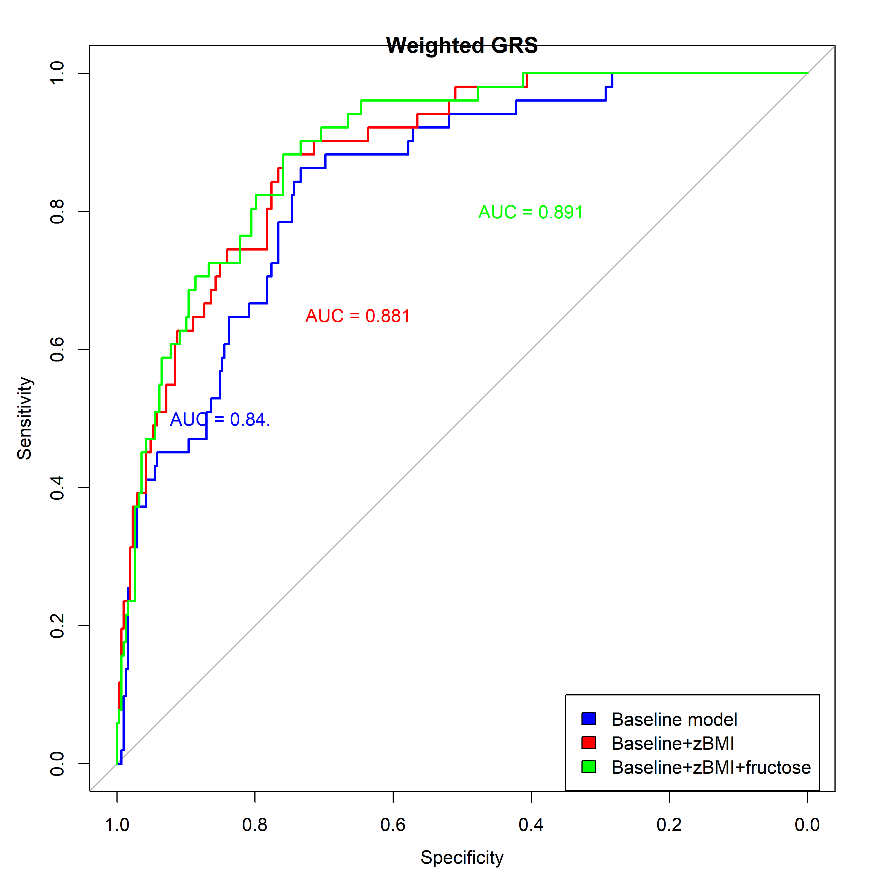


**Supplementary figure 6**. Receiver operating characteristics (ROC) curves of the unweighted (uGRS) and weighted (wGRS) genetic risk scores adjusted by principal components analyses, sex and age with BMI-z score and pure fructose from non-natural foods or birth weight or physical activity or sodium consumption added to the model. Areas under curves (AUC) are indicated. The straight line represents the ROC expected by chance only.

#
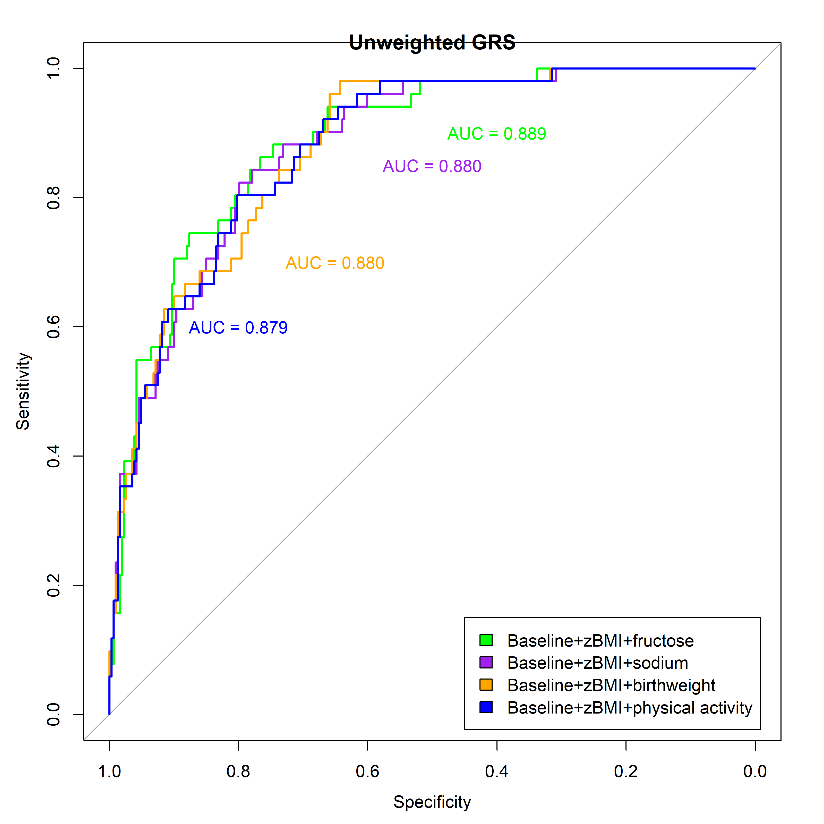


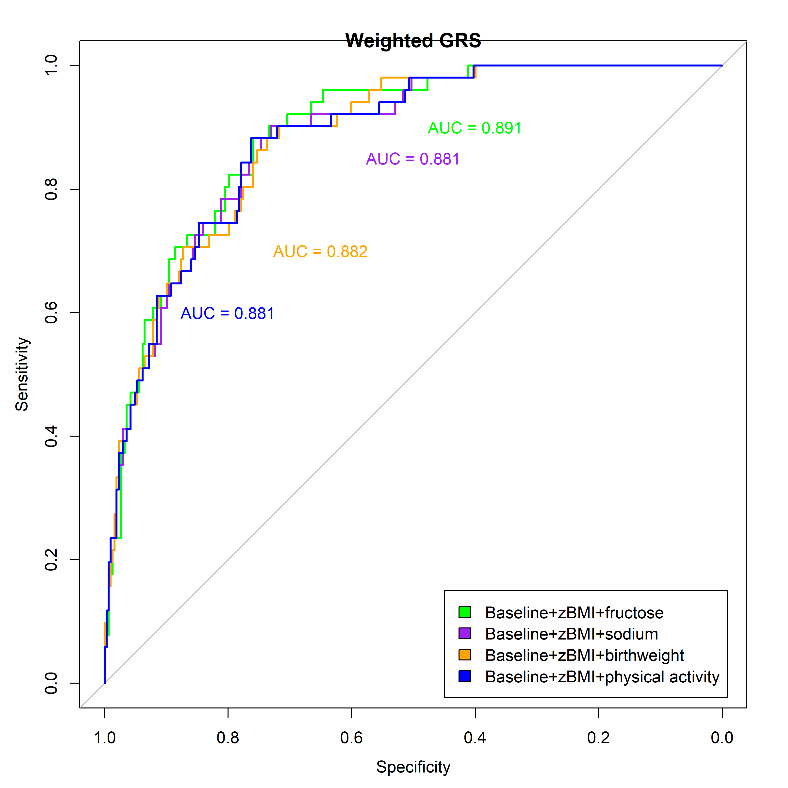


# List of HELENA study group

**HELENA Study Group**

**Co-ordinator:** Luis A. Moreno.

**Core Group members:** Luis A. Moreno, Fréderic Gottrand, Stefaan De Henauw, Marcela González-Gross, Chantal Gilbert.

**Steering Committee:** Anthony Kafatos (President), Luis A. Moreno, Christian Libersa, Stefaan De Henauw, Sara Castelló, Fréderic Gottrand, Mathilde Kersting, Michael Sjöstrom, Dénes Molnár, Marcela González-Gross, Jean Dallongeville, Chantal Gilbert, Gunnar Hall, Lea Maes, Luca Scalfi.

**Project Manager:** Pilar Meléndez.

1. **Universidad de Zaragoza (Spain)**

Luis A. Moreno, José A. Casajús, Jesús Fleta, Gerardo Rodríguez, Concepción Tomás, María I. Mesana, Germán Vicente-Rodríguez, Adoración Villarroya, Carlos M. Gil, Ignacio Ara, Juan Fernández Alvira, Gloria Bueno, Olga Bueno, Juan F. León, Jesús Mª Garagorri, Idoia Labayen, Iris Iglesia, Silvia Bel, Luis A. Gracia Marco, Theodora Mouratidou, Alba Santaliestra-Pasías, Iris Iglesia, Esther González-Gil, Pilar De Miguel-Etayo, Cristina Julián, Mary Miguel-Berges, Isabel Iguacel, Azahara Rupérez, Miguel Seral-Cortes and Gloria Pérez-Gimeno.

1. **Consejo Superior de Investigaciones Científicas (Spain)**

Ascensión Marcos, Julia Wärnberg, Esther Nova, Sonia Gómez, Ligia Esperanza Díaz, Javier Romeo, Ana Veses, Belén Zapatera, Tamara Pozo, David Martínez.

1. **Université de Lille 2 (France)**

Laurent Beghin, Christian Libersa, Frédéric Gottrand, Catalina Iliescu, Juliana Von Berlepsch.

1. **Research Institute of Child Nutrition Dortmund, Rheinische Friedrich-Wilhelms-Universität Bonn (Germany)**

Mathilde Kersting, Wolfgang Sichert-Hellert, Ellen Koeppen.

1. **Pécsi Tudományegyetem (University of Pécs) (Hungary)**

Dénes Molnar, Eva Erhardt, Katalin Csernus, Katalin Török, Szilvia Bokor, Mrs. Angster, Enikö Nagy, Orsolya Kovács, Judit Répasi.

1. **University of Crete School of Medicine (Greece)**

Anthony Kafatos, Caroline Codrington, María Plada, Angeliki Papadaki, Katerina Sarri, Anna Viskadourou, Christos Hatzis, Michael Kiriakakis, George Tsibinos, Constantine Vardavas, Manolis Sbokos, Eva Protoyeraki, Maria Fasoulaki.

1. **Institut für Ernährungs- und Lebensmittelwissenschaften – Ernährungphysiologie. Rheinische Friedrich Wilhelms Universität (Germany)**

Peter Stehle, Klaus Pietrzik, Marcela González-Gross, Christina Breidenassel, Andre Spinneker, Jasmin Al-Tahan, Miriam Segoviano, Anke Berchtold, Christine Bierschbach, Erika Blatzheim, Adelheid Schuch, Petra Pickert.

1. **University of Granada (Spain)**

Manuel J. Castillo, Ángel Gutiérrez, Francisco B Ortega, Jonatan R Ruiz, Enrique G Artero, Vanesa España, David Jiménez-Pavón, Palma Chillón, Cristóbal Sánchez-Muñoz, Magdalena Cuenca

1. **Istituto Nazionalen di Ricerca per gli Alimenti e la Nutrizione (Italy)**

Davide Arcella, Elena Azzini, Emma Barrison, Noemi Bevilacqua, Pasquale Buonocore, Giovina Catasta, Laura Censi, Donatella Ciarapica, Paola D'Acapito, Marika Ferrari, Myriam Galfo, Cinzia Le Donne, Catherine Leclercq, Giuseppe Maiani, Beatrice Mauro, Lorenza Mistura, Antonella Pasquali, Raffaela Piccinelli, Angela Polito, Romana Roccaldo, Raffaella Spada, Stefania Sette, Maria Zaccaria.

1. **University of Napoli "Federico II" Dept of Food Science (Italy)**

Luca Scalfi, Paola Vitaglione, Concetta Montagnese.

1. **Ghent University (Belgium)**

Ilse De Bourdeaudhuij, Stefaan De Henauw, Tineke De Vriendt, Lea Maes, Christophe Matthys, Carine Vereecken, Mieke de Maeyer, Charlene Ottevaere, Inge Huybrechts.

1. **Medical University of Vienna (Austria)**

Kurt Widhalm, Katharina Phillipp, Sabine Dietrich, Birgit Kubelka
Marion Boriss-Riedl.

1. **Harokopio University (Greece)**

Yannis Manios, Eva Grammatikaki, Zoi Bouloubasi, Tina Louisa Cook, Sofia Eleutheriou, Orsalia Consta, George Moschonis, Ioanna Katsaroli, George Kraniou, Stalo Papoutsou, Despoina Keke, Ioanna Petraki, Elena Bellou, Sofia Tanagra, Kostalenia Kallianoti, Dionysia Argyropoulou, Stamatoula Tsikrika, Christos Karaiskos.

1. **Institut Pasteur de Lille (France)**

Jean Dallongeville, Aline Meirhaeghe.

1. **Karolinska Institutet (Sweden)**

Michael Sjöstrom, Jonatan R Ruiz, Francisco B. Ortega, María Hagströmer, Anita Hurtig Wennlöf, Lena Hallström, Emma Patterson, Lydia Kwak, Julia Wärnberg, Nico Rizzo.

1. **Asociación de Investigación de la Industria Agroalimentaria (Spain)**

Jackie Sánchez-Molero, Sara Castelló, Elena Picó, Maite Navarro, Blanca Viadel, José Enrique Carreres, Gema Merino, Rosa Sanjuán, María Lorente, María José Sánchez.

1. **Campden BRI (United Kingdom)**

Chantal Gilbert, Sarah Thomas, Elaine Allchurch, Peter Burgess.

1. **SIK - Institutet foer Livsmedel och Bioteknik (Sweden)**

Gunnar Hall, Annika Astrom, Anna Sverkén, Agneta Broberg.

1. **Meurice Recherche & Development asbl (Belgium)**

Annick Masson, Claire Lehoux, Pascal Brabant, Philippe Pate, Laurence Fontaine.

1. **Campden & Chorleywood Food Development Institute (Hungary)**

Andras Sebok, Tunde Kuti, Adrienn Hegyi.

1. **Productos Aditivos SA (Spain)**

Cristina Maldonado, Ana Llorente.

1. **Cárnicas Serrano SL (Spain)**

Emilio García.

1. **Cederroth International AB (Sweden)**

Holger von Fircks, Marianne Lilja Hallberg, Maria Messerer

1. **Lantmännen Food R&D (Sweden)**

Mats Larsson, Helena Fredriksson, Viola Adamsson, Ingmar Börjesson.

1. **European Food Information Council (Belgium)**

Laura Fernández, Laura Smillie, Josephine Wills.

1. **Universidad Politécnica de Madrid (Spain)**

Marcela González-Gross, Raquel Pedrero-Chamizo, Agustín Meléndez, Jara Valtueña, David Jiménez-Pavón, Ulrike Albers, Pedro J. Benito, Juan José Gómez Lorente, David Cañada, Alejandro Urzanqui, Rosa María Torres, Paloma Navarro.
